# Sleep Paralysis, “The Ghostly Bedroom Intruder” and Out-of-Body Experiences: The Role of Mirror Neurons

**DOI:** 10.3389/fnhum.2017.00092

**Published:** 2017-02-28

**Authors:** Baland Jalal, Vilayanur S. Ramachandran

**Affiliations:** ^1^Behavioural and Clinical Neuroscience Institute and Department of Psychiatry, University of CambridgeCambridge, UK; ^2^Center for Brain and Cognition, University of California at San DiegoLa Jolla, CA, USA

**Keywords:** sleep paralysis, mirror neurons, hallucinations, out of body experience, REM sleep, hypnopompic hallucinations, hypnogoic

Rapid eye movement (REM) sleep—for good reasons—is referred to as *paradoxical sleep*: our blood pressure, heart rate, and breathing become elevated. And electroencephalography (EEG) recordings show a peculiar, lower voltage, and mixed frequency pattern (La Berge et al., 1981; Horne, 2013). In fact, the firing pattern of most neurons during REM sleep resemble those of wakefulness—and in some cases neurons fire in even more intense bursts (e.g., in the pons, lateral geniculate nucleus, and occipital cortex), than when we're awake (Kandel et al., 2000). This is not all too surprising, as we have our most vivid and emotionally-charged dreams during REM sleep, often involving a complex story plot. In order for us not to act out these dreams—and potentially hurt ourselves—our brain has an ingenious solution: it leaves us temporarily paralyzed from head to toe. This paralysis (postural atonia) is triggered by the pons (including the pontine reticular formation) and ventromedial medulla that suppress skeletal muscle tone during REM sleep—via inhibition of motor neurons in the spinal cord; through neurotransmitters GABA and glycine (Brooks and Peever, 2012; Jalal and Hinton, 2013).

Occasionally, perceptual activation occurs (we start to wake up mentally), while under the “spell” of REM paralysis. The result is a curious condition called sleep paralysis (SP), where the person is left “trapped”—unable to move or speak upon falling asleep or upon awakening (Hobson, 1995; Jalal et al., 2014a). Intriguingly during SP, the sensory system is clear, and ocular, and respiratory movements remain intact, culminating in a state of semi-consciousness coupled with bodily paralysis (Jalal and Hinton, 2013). While once thought to only arise in the context of narcolepsy—a rare autoimmune sleep disorder affecting <1% of the population (Jalal, 2016)—we now know that 20% of the general population have SP episodes (Sharpless and Barber, 2011; Jalal and Hinton, 2013).

During SP, the vivid—and sometimes terrifying—dreams of REM sleep (REM mentation) can spill over into emerging wakefulness (Jalal and Hinton, 2015). Hypnogogic or hypnopompic hallucinations occur in all sensory modalities, and include out-of-body experiences (OBE), and sensing and seeing the presence of menacing intruders in one's bedroom (Jalal and Hinton, 2013; Jalal and Ramachandran, 2014; Jalal et al., 2014b, 2015).

We have proposed that a functional disturbance of the (right) parietal cortex may give rise to the common “bedroom intruder” hallucination seen during SP (Jalal and Ramachandran, 2014). As described, the absence of afferent sensory signals might cause this disturbance of “body image”; implicating regions such as the right superior parietal lobule (SPL) and the temporoparietal junction (TPJ)—critical for the construction of a neural representation of the body. Essential to this hypothesis, is the hallucinated projection of a genetically hardwired body-map (homunculus) due to conflicting (efferent and afferent) neural conduction. This hypothesis is broadly consistent with the finding that disrupting the TPJ using focal electrical stimulation can induce the feeling of an illusory “other” shadow-like person mimicking one's body postures (Arzy et al., 2006); and that hyperactivity in the temporoparietal cortex of schizophrenics can lead to the misattribution of their own actions to others (Farrer et al., 2004).

We further evoke the mirror neuron system (MNS) as crucial in giving rise to this “intruder” hallucination. Neurons in area V5 of the premotor cortex fire when you make volitional movements. Intriguingly, a subset of them (10%), fire even when you merely watch another person performing the action. These neurons—dubbed mirror neurons—allow higher centers to say in effect “the same cells are firing as would fire if I were about to reach out for the peanut—so that's what the other person is *intending* to do” (Rizzolatti et al., 1996, 2001). Circuits performing analogous computations may be involved in reading the higher order intentions that are required for constructing a *theory of mind* (ToM), but this is still a matter of some debate.

The MNS allows you to temporarily detach yourself from your body and “see” the world from another person's vantage point. In other primates, this requires the physical presence of another individual—whereas, in humans, it might be that the MNS is sufficiently well connected that it allows you a virtual point of view (i.e., imagine what you would be seeing if you were in the other person's place). However, even though you temporarily see the world from another's location—you don't literally leave your body (i.e., you don't have an out-of-body experience [OBE]). This is because the MNS has multiple outputs, which are powerfully modulated by two sources. First, sensory afferents from the body—and, second—prefrontal cortex. The triadic interaction between MNS, prefrontal cortex (anterior to V5), and sensory feedback results in a dual representation—a feeling that you are “out there” looking at someone else's actions—while at the same time being fully anchored, here, and now in your own body (Ramachandran, 2012).

This interaction involves a convergence of inputs in the right SPL, and their target zones in V5. Not surprisingly, damage to the prefrontal cortex sometimes results in echopraxia—i.e., miming what somebody near is doing. Analogously, the massive deafferentation of sensory input during SP would lead to a similar disinhibition of the MNS and its propensity to project its body into another individual—if you are a chimp—or another virtual body, if you are a human. A disturbance of these interactions would lead to the more florid manifestations of an alien abductor, bedroom intruder, or mysterious other—seen so frequently during SP. In addition, we suggest that OBEs during SP, likewise result from the massive deafferentation that occurs during REM sleep paralysis. These ideas could be explored using neuroimaging, to examine the selective activation of brain regions associated with mirror neuron activity, when the individual is hallucinating an intruder or having an OBE during SP.

## Author contributions

BJ and VR came up with the intellectual content of the article, and wrote up the article.

### Conflict of interest statement

The authors declare that the research was conducted in the absence of any commercial or financial relationships that could be construed as a potential conflict of interest. The reviewer LP and handling Editor declared their shared affiliation, and the handling Editor states that the process nevertheless met the standards of a fair and objective review.
